# Systematic reviews of low-frequency repetitive transcranial magnetic stimulation on cognition and epileptiform discharge in patients with epilepsy

**DOI:** 10.7717/peerj.20637

**Published:** 2026-02-09

**Authors:** Simin Xu, Shufan Li, Fen Yu, Chen Wei, Feng Ding, Xing Wang, Shihang Lin

**Affiliations:** 1 School of Physical Education, Shanghai University of Sport, Shanghai, China; 2 College of Sports Science, Shenyang Normal University, Shenyang, China

**Keywords:** Low-frequency repetitive transcranial magnetic stimulation, Epilepsy, Cognitive function, Systematic reviews, Epileptiform discharge

## Abstract

**Background:**

This systematic review investigates the efficacy of low-frequency repetitive transcranial magnetic stimulation (LF-rTMS) in improving cognitive function and reducing epileptiform discharges in patients with epilepsy. It further examines whether patient age moderates the treatment effect. Additionally, the review evaluates whether intervention parameters, including duration, frequency, session time, and stimulation site, positively influence the improvement of cognition and epileptiform discharges.

**Methods:**

Seven databases were searched: Embase, Web of Science, PubMed, The Cochrane Library, Wanfang, VIP, and China National Knowledge Infrastructure. The search period was from database inception to September 2025. Two researchers independently screened the literature to identify randomized controlled trials (RCTs) that investigated the effects of TMS on cognition in patients with epilepsy. Quality assessment was performed using PEDro, and Meta-analysis and publication bias were tested using RevMan 5.4.1 and Stata 17.0, respectively. The quality of evidence for outcome indicators was evaluated using GRADEPro software. The standardized mean difference (SMD) and 95% CI were used as effect size statistics.

**Results:**

This meta-analysis included 12 randomized controlled trials (*n* = 1,289 patients). Pooled results demonstrated that low-frequency rTMS (LF-rTMS) significantly improved cognitive function (SMD = 1.22, 95% CI [0.87–1.56], *P* < 0.0001; *n* = 861) and reduced epileptiform discharges (SMD = −0.68, 95% CI [−0.98 to −0.37], *P* < 0.00001; *n* = 428). Subgroup analyses identified key parameters associated with optimal outcomes. For cognitive improvement, greater effect sizes were observed in patients aged 45–60 years, with an LF-rTMS protocol of 1 Hz stimulation targeting the epileptogenic focus, session time >20 min, intervention duration >4 weeks, and frequency of ≤7 sessions/week, particularly when combined with levetiracetam. Conversely, a greater reduction in epileptiform discharges was associated with younger age (<45 years) and shorter intervention duration (≤1 week).

**Conclusion:**

The included studies (average PEDro = 6.3) had moderate quality, limited by incomplete blinding/allocation reporting. Heterogeneity stemmed from intervention parameters (site, duration, frequency, medications). No significant publication bias was observed. Evidence quality was high for cognition and moderate for epileptiform discharge. LF-rTMS effectively improves both outcomes, influenced by treatment protocols, supporting its clinical use in the management and treatment of epilepsy.

**Other:**

This study has been registered on PROSPERO No. CRD42024593502. Registration Platform: www.crd.york.ac.uk.

## Introduction

Epilepsy is a brain network disorder that is fundamentally caused by abnormal synchronized neuronal discharge. It typically presents with epileptic seizures and cognitive impairment (Lu & Yu, 2022). Seizures are characterized by their transient, recurrent, stereotyped, and paroxysmal nature and are frequently accompanied by epileptiform discharges (EDs) (Fisher et al., 2017). The World Health Organization recognizes epilepsy as one of the five major mental health disorders that receives substantial global attention. Epidemiological data indicate that approximately 50 million individuals worldwide have epilepsy, with more than 10 million cases reported in China (Zhou & Lin, 2022). Epilepsy can lead to cognitive dysfunction, disturbances of consciousness, and generalized muscle spasms, severely impairing patients’ quality of life (Wang et al., 2024). The disease is difficult to cure and is prone to relapse, imposing substantial economic burdens and social pressure on patients. Therefore, effectively improving cognitive function and abnormal brain discharges in patients with epilepsy and alleviating their suffering remain urgent issues to be addressed.

Repetitive transcranial magnetic stimulation (rTMS) is a noninvasive neuromodulation technxique used to treat neurological diseases. It has become a hotspot in adjuvant epilepsy therapy due to its advantages of being noninvasive, safe, well-tolerated, and easy-to-operate nature (Chen & Cui, 2022). Evidence suggests that rTMS may exert therapeutic effects in epilepsy by modulating cortical excitability, enhancing synaptic plasticity, and regulating neurotransmitter systems (Wang et al., 2024; Wu et al., 2023). High-frequency rTMS can increase cortical excitability, whereas low-frequency rTMS can inhibit cortical function and reduce excitability (Joo et al., 2007; Xiong, Zheng & Wang, 2022). At the same time, it is generally believed that an imbalance between excitatory and inhibitory activities in the brain, which leads to increased cortical excitability, plays an important role in the pathophysiology of epilepsy (Bauer et al., 2018). Therefore, most studies on epilepsy use low-frequency repetitive transcranial magnetic stimulation (LF-rTMS) at a frequency of 0.2–1 Hz. A review of previous studies found that there is no consensus on the stimulation frequency and site for using LF-rTMS to treat cognitive function in patients with epilepsy. Hu et al. (2023) and Wang et al. (2024) found that a stimulation frequency of 0.5 Hz can improve the EEG and cognitive function more effectively than 0.3 and 1 Hz. Zhang (2020) believes that 1 Hz low-frequency repetitive transcranial magnetic stimulation is more effective for the recovery of cognitive function in epilepsy patients. In studies by Fregni et al. (2006) and Hu et al. (2024), LF-rTMS at 0.5 and 1 Hz significantly reduced the number of spikes in patients’ interictal spikes or spikes, thereby improving epileptiform discharge. However, Cantello et al. (2007) showed that only nearly one-third of epilepsy patients had a significant decrease in spikes after LF-rTMS intervention at 0.3 Hz.

In summary, the efficacy of low-frequency repetitive transcranial magnetic stimulation (LF-rTMS) in improving cognitive function and abnormal epileptiform discharges in patients with epilepsy is still controversial. Moreover, the optimal stimulation site and frequency of LF-rTMS in patients with epilepsy remain uncertain, and differences in therapeutic efficacy have been observed across varying intensities and intervention durations. Previous systematic reviews have largely overlooked the efficacy of LF-rTMS in improving cognitive function and ED in epilepsy, and few have conducted stratified analyses to determine optimal intervention protocols. Therefore, this study aimed to systematically evaluate the effects of LF-rTMS on cognitive function and ED in patients with epilepsy, identify the optimal intervention protocol, address the limitations of existing systematic reviews, and provide evidence-based support for clinical practice in epilepsy.

## Data and methods

This study followed the requirements of the Meta-Analysis PRISMA Writing Guidelines (Page et al., 2021) for the selection and use of research methods and was registered in the International Prospective Register of Systematic Reviews (PROSPERO) (No. CRD42024593502).

### Study structure

This study is grounded in the International Classification of Functioning, Disability, and Health (ICF) framework (Qiu et al., 2020). It analyzes patient characteristics such as age, stimulation frequency, intervention duration, and treatment duration, as well as the effects of low-frequency repetitive transcranial magnetic stimulation (LF-rTMS) on cognitive function and abnormal epileptiform discharges in epilepsy, from the perspective of changes in these outcomes. The PICOS framework guiding this systematic review is shown in Table 1.

**Table 1:** PICOS architecture of LF-rTMS intervention for cognition and ED in epilepsy patients.

Population	Intervention	Comparison	Outcome	Study design
Epileptic	Type of intervention	With or without low-frequency repetitive transcranial magnetic stimulation therapy	Cognitive functions	Randomised controlled trial
			Mental functions b1	
Age ≥ 18 years	Low-frequency repetitive transcranial magnetic stimulation + conventional medication	Prescription of different interventions	Attention b140	(Randomized controlled trial)
	Prescription for intervention		Memory b144	
	Stimulation frequency		Thinking b160	
	Intervention time		Higher level cognitive functions b164	
	Intervention period		Electrical discharge	
	Intervention frequency		Nervous system s1	
	Stimulus area		Brain s110	
	Combination of drugs			

### Search strategy

Two researchers (L.S.F and Y.F) independently searched the following databases: Embase, Web of Science, PubMed, The Cochrane Library, Wanfang, VIP, and China National Knowledge Infrastructure for randomized controlled trials (RCTs) that investigated the effect of repetitive transcranial magnetic stimulation on cognition and ED in patients with epilepsy. The search period was from the date each database was first established to September 2025. Reference lists of retrieved articles were also manually searched for additional studies. The literature search strategy is presented in Table 2.

**Table 2:** Literature search strategy.

Comprehensive database	Search step
PubMed and The Cochrane Library search strategies	#1“Epilepsy”[MeSH Terms] OR “seizure disorder”[Title/Abstract] OR “seizure disorders”[Title/Abstract] OR “epilepsy cryptogenic”[Title/Abstract] OR “cryptogenic epilepsies”[Title/Abstract] OR “cryptogenic epilepsy”[Title/Abstract]
	#2 “Transcranial Magnetic Stimulation”[MeSH Terms] OR “magnetic stimulation transcranial”[Title/Abstract] OR “stimulation transcranial magnetic”[Title/Abstract] OR “transcranial magnetic stimulations”[Title/Abstract]
	#3 “Cognition”[MeSH Terms] OR “cognitive impairment”[Title/Abstract] OR “cognitive capacity”[Title/Abstract] OR “cognitive impairment”[Title/Abstract] OR “Memory”[Title/Abstract] OR “Attention”[Title/Abstract] OR “Language”[Title/Abstract] OR “executive function”[Title/Abstract] OR “Electroencephalography”[MeSH Terms] OR “EEG”[Title/Abstract] OR “Electroencephalogram”[Title/Abstract] OR “Electroencephalograms”[Title/Abstract] OR “epileptiform discharges”[Title/Abstract] OR “epileptic discharge”[Title/Abstract] OR “Electroencephalography”[Title/Abstract] OR “EEG”[Title/Abstract] OR “Electroencephalogram”[Title/Abstract] OR “Electroencephalograms”[Title/Abstract]
	#4 Randomized controlled trial [Publication Type] OR “Randomized” [Title/Abstract] OR “controlled” [Title/Abstract] OR “Trial” [Title/Abstract]
	#5 #1 AND #2 AND #3 AND #4
Web of Science search strategies	#1 TS=(Epilepsy OR ‘seizure disorder’ OR ‘seizure disorders’ OR ‘epilepsy cryptogenic’ OR ‘cryptogenic epilepsies’ OR ‘cryptogenic epilepsy’’) and Preprint Citation Index (Exclude–Database)
	#2 TS=(‘Transcranial Magnetic Stimulation’ OR ‘magnetic stimulation transcranial’ OR ‘stimulation transcranial magnetic’ OR ‘transcranial magnetic stimulations’) and Preprint Citation Index (Exclude–Database)
	#3 TS=(Cognitive OR Memory OR Attention OR Language OR ‘Executive function’ OR ‘epileptiform discharges’ OR ‘epileptic discharge’ OR Electroencephalography OR EEG OR Electroencephalogram OR Electroencephalograms)
	#4 TS=(“Randomized controlled trial” OR “Randomized” OR “Controlled” OR “Trial”)
	#5 #1 AND #2 AND #3 AND #4
Embase search strategy	#1 “epilepsy” [exp] OR “acute epilepsy” [ab,ti] OR “attack, epileptic”[ab,ti] OR “cerebral seizure, epileptic” [ab,ti] OR “chronic epilepsy” [ab,ti] OR “comitial disease” [ab,ti] OR “convulsion, epileptic” [ab,ti]
	#2 “transcranial magnetic stimulation” [exp] OR “magnetic stimulation, transcranial” [ab,ti] OR “repetitive transcranial magnetic stimulation” [ab,ti]
	#3 “cognition” [exp] OR “cognitive accessibility” [ab,ti] OR “cognitive function” [ab,ti] OR “cognitive structure” [ab,ti] OR “cognitive capacity” [ab,ti] OR “epileptic discharge” [exp] OR “discharge, epileptic” [ab,ti] OR “discharge, epileptoid” [ab,ti] OR “discharge, hypersynchronous neuronal” [ab,ti] OR “discharge, neuronal” [ab,ti]
	#4 “Randomized controlled trial” [exp] OR “Randomized” [ab,ti] OR “Controlled” [ab,ti] OR “Trial” [ab,ti]
	#5 #1 AND #2 AND #3 AND #4
China knowledge network search strategy	主题=(癫痫 + 癫痫病 + 癫痫发作 + 癫痫样) AND主题=(经颅磁刺激 + 重复性经颅磁刺激 + ‘经颅磁刺激(rtms)’ + ‘经颅磁刺激(tms))AND主题=(癫痫样 + 癫痫样放电 + 癫痫样脑电图 + 癫痫样棘波 ＋ 认知 + 认知功能 + 认知能力 ＋ 执行功能 ＋ 注意力 ＋ 记忆)
Wanfang, Wipu search strategy	主题=(癫痫 OR 癫痫病 OR 癫痫发作 OR 癫痫样) AND主题=(经颅磁刺激 OR 重复性经颅磁刺激)AND主题=(癫痫样 OR 癫痫样放电 OR 癫痫样脑电图 OR 癫痫样棘波 OR 认知 OR 认知功能 OR 认知能力 OR 执行功能 OR 注意力 OR 记忆)

### Literature inclusion and exclusion criteria

#### Inclusion criteria

(1) Participants who met the diagnostic criteria for epilepsy by Neurology (7th edition) (Jia, 2013), the Criteria for the Diagnosis of Clinical Diseases and the Judgment of Efficacy (Wang, 2010), and the International League Against Epilepsy’s 2001 Recommendations for the Diagnosis of Epilepsy and Epileptic Syndromes (Engel, 2001), and who were diagnosed with epilepsy based on abnormal neuronal discharges on electroencephalography, such as the appearance of spikes, sharp waves, and spike-and-wave complexes or sharp-wave-and-slow-wave complexes (Hasan & Tatum, 2021). Studies enrolling patients with focal or generalized epilepsy types were eligible to evaluate the broad applicability of LF-rTMS. (2) The diagnosis was supported by EEG evidence of epileptiform discharges (EDs), defined as the presence of spikes, sharp waves, or spike-wave complexes, which are biomarkers of cortical hyperexcitability. (3) In randomized controlled trials, patients in the control group received routine medical treatment combined with sham stimulation, while those in the intervention group received identical routine treatment supplemented with active low-frequency rTMS (≤1 Hz). (4) The specific number of patients in the treatment and control groups is determined. (5) At least one outcome measure (seizure frequency, cognition, epileptiform electroencephalogram (EEG)) is reported before and after stimulation.

#### Exclusion criteria

(1) Review articles, case reports, commentaries, letters, and conference reports, as these publication types typically lack original quantitative data necessary for systematic analysis; (2) participants who had previously undergone epilepsy surgery, to minimize potential confounding effects on treatment response; (3) studies with inadequately described rTMS stimulation protocols were excluded. We attempted to contact the corresponding authors for clarification; however, no responses were received, and the missing details could not be supplemented; (4) unavailable full texts, which prevented adequate assessment of methodological quality and data extraction.

### Literature screening, data extraction and quality assessment

#### Literature screening and data extraction

The identified records were imported into EndNote X9 for duplicate removal. Two investigators (D.F. and C.W.) independently conducted study selection, data extraction, and quality assessment under double-blind conditions, with both unaware of each other’s evaluations. The extracted data from the eligible studies were entered into RevMan 5.4.1 software (The Cochrane Collaboration, https://test-training.cochrane.org/online-learning/core-software-cochrane-reviews/review-manager-revman/download-revman-5) and independently cross-verified by the same two authors. Any discrepancies during study selection, data extraction, or quality scoring were initially discussed between D.F. and C.W. to reach a consensus on the final decision. If agreement could not be achieved, a third reviewer (Y.F.) was consulted to perform an independent assessment and make a final decision. This structured approach ensured the consistent, transparent, and unbiased resolution of all disagreements. Data extraction included the first author, publication year, country of origin, baseline characteristics (age, sex, disease duration), interventions, and outcome measures. Cognitive function was assessed by extracting scores from standardized tools, chiefly the Mini-Mental State Examination (MMSE) and the Montreal Cognitive Assessment (MoCA), the latter of which is more sensitive in detecting mild cognitive impairment. In both instruments, higher scores indicate superior cognitive function.

#### Quality assessment

The methodological quality of the included studies was evaluated using the PEDro scale (Ludyga et al., 2020), which consists of ten criteria: random allocation, allocation concealment, baseline comparability, blinding of participants, blinding of therapists, blinding of outcome assessors, participation rate >85%, intention-to-treat analysis, between-group statistical comparisons, and point estimates with variability. Each satisfied criterion was awarded one point, and the unmet criteria were scored zero. The maximum score on the scale is 10. Scores <4 indicate low quality, 4–5 indicate moderate quality, 6–8 indicate good quality, and 9–10 indicate high quality. Only studies of moderate or higher quality were included.

The GRADEpro evidence grading system was also used to evaluate the quality of the evidence for outcome indicators (Page et al., 2021), with the quality of evidence for each outcome indicator divided into four categories: high, moderate, low, and very low. Two researchers (D.F. and C.W.) independently assessed the quality scores of the included studies under blinding conditions, where each reviewer was unaware of the other’s evaluations during data extraction and quality scoring. Any discrepancies in scoring were discussed between the two reviewers to reach a consensus. If an agreement could not be reached, a third researcher (Y.F.) performed an independent assessment of the disputed items. The final score was determined based on the evaluation of the third researcher. This structured procedure was implemented to ensure objectivity, consistency, and transparency in the quality appraisal process.

### Data processing

Heterogeneity was assessed using RevMan 5.4.1 based on the sample size and the mean and standard deviation of the pre- and post-intervention improvement values for all included outcome indicators. All the outcome indicators were continuous variables. For indicators measured using the same method and unit, the mean difference (MD) was calculated; for those measured using different methods or units, the standardized mean difference (SMD) was applied. Heterogeneity was evaluated using *P* values and I^2^ statistics. A threshold of *P* < 0.05 and I^2^ > 50% indicated significant heterogeneity, in which case a random-effects model was applied; otherwise, a fixed-effects model was used. The 95% confidence interval (95% CI) was reported as the effect estimate. Publication bias was assessed using Stata version 17.0.

## Results

### Results of literature search

**Figure 1:**
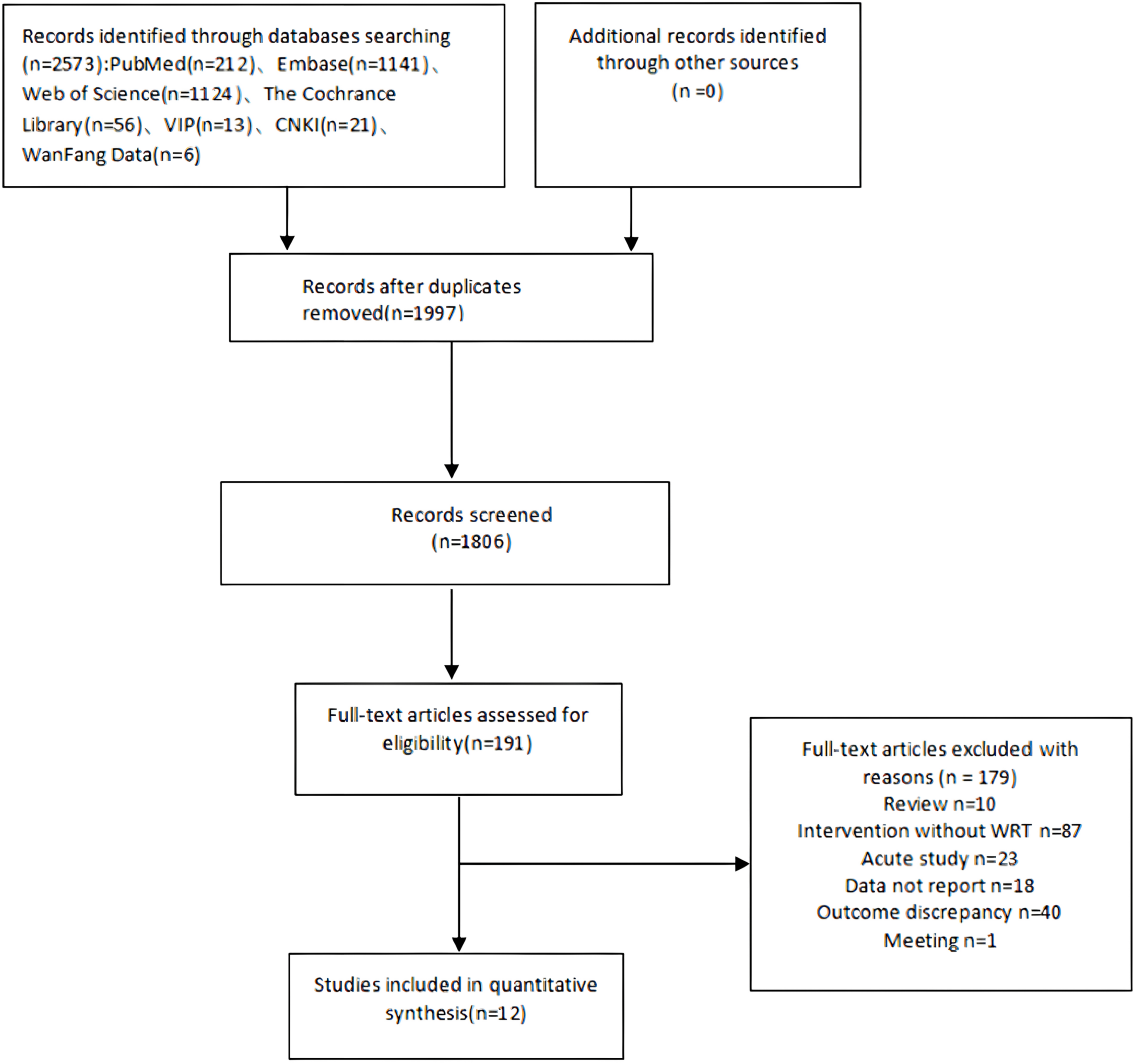
Flowchart of literature screening.

A total of 2,573 related studies were retrieved, and 12 articles were finally included (Cantello et al., 2007; Du & Zhao, 2021; Fregni et al., 2006; Wang, Zhao & Du, 2022; Zhao, Zheng & Ma, 2018; Hu et al., 2024; Geng, 2022; Lai et al., 2018; Hu et al., 2023; Huang, 2018; Zhang, 2020; Gao, 2020; Zhao, 2018). See Fig. 1.

### Basic information about the included literature

This study included 12 publications comprising 18 studies (Cantello et al., 2007; Du & Zhao, 2021; Fregni et al., 2006; Wang, Zhao & Du, 2022; Zhao, Zheng & Ma, 2018; Hu et al., 2024; Geng, 2022; Lai et al., 2018; Hu et al., 2023; Huang, 2018; Zhang, 2020; Gao, 2020; Zhao, 2018), involving a total of 1,289 participants. Of these, 629 participants were assigned to intervention groups and 660 to control groups. All participants were clinically diagnosed with epilepsy according to the diagnostic criteria of the International League Against Epilepsy (ILAE). The included studies were published between 2006 and 2024. In all studies, the intervention groups received rTMS, whereas the control groups received conventional pharmacotherapy or sham stimulation. The included studies reported intervention duration, duration, and frequency of the intervention. Specifically, the intervention duration of rTMS ranged from 2 to 12 weeks, with a frequency of 1–2 sessions per day, and each session lasted 20–90 min. The basic characteristics of the included studies are summarized in Table 3.

**Table 3:** Basic information of the included literature.

Inclusion of studies	Country/Area	Sample size	Gender (M/F)		Age (years)		Course of disease		Intervention	Intervention dose	Assessment tools
		(T/C)	(T/C)		(T/C)		(T/C)		(T/C)		
Fregni et al. (2006)	America	12/9	Total 9/12		21.3 ± 6.4	22.7 ± 10.3	——	——	A+B/A+C	2 h/dose, 5 times a day for 5 days, 2 months follow-up	①②③
Du & Zhao (2021)	China	33/33	18/15	16/17	49.89 ± 8.56	49.78 ± 9.56	5.69 ± 3.56	6.05 ± 3.15	A+B/A	30 min/dose, 1 day, 1 month total	④
Lai et al. (2018)	China	30/30	——	——	33.48 ± 2.02	33.54 ± 2.05	4.09 ± 0.98	4.12 ± 1.02	A+B/A	2 times per week, 8 weeks as a course of treatment, 3 courses of treatment	⑤
Hu et al. (2023)	China	32/32	17/15	16/16	65.23 ± 5.31	65.31 ± 5.35	——	——	A+B/A	6 times a week for 1 month	⑤
Huang (2018)	China	28/28	13/15	12/16	43.0 ± 6.5	41.5 ± 6.7	——	——	A+B/A	20 min/dose, 2 times a day for 2 weeks	⑤
Hu et al. (2023)	China	32/32	18/14	16/16	65.31 ± 5.35	65.31 ± 5.35	——	——	A+B/A	6 times a week for 1 month	⑤
Geng (2022)	China	41/41	24/17	26/15	38.91 ± 5.36	38.74 ± 5.20	4.37 ± 1.10	4.31 ± 1.09	A+B/A+C	Stimulation 10 s, Interval 5 s, Repeat stimulation 100 times, 5 d/week for 3 months.	④
Wang, Zhao & Du (2022)	China	40/40	16/24	17/23	45.04 ± 8.47	42.86 ± 7.68	28.11 ± 6.37 years	26.45 ± 6.19 years	A+B/A	22 min/d, 1x/d, total 8 weeks	⑤
Zhao, Zheng & Ma (2018)	China	57/57	32/25	33/24	47.52 ± 11.26	48.19 ± 10.43	6.92 ± 1.67 years	7.45 ± 1.81	D+B/D	20 min/dose, 2 times a day for 4 weeks	④⑥
Gao (2020)	China	53/53	27/26	25/28	49.28 ± 4.32	46.71 ± 5.57	——	——	D+B/D	20 min/dose, 1 day for 4 weeks	④
Hu et al. (2024)	China	68/53	31/22	34/34	67.68 ± 13.48	64.56 ± 12.81	——	——	A+B/A	40 min/dose, 1 week continuous treatment	⑤⑥
Zhao (2018)	China	60/60	28/32	27/33	5.14 ± 1.47	4.86 ± 1.68	——	——	A+B/A	Maximum pulse volume 1,000 pulses/d for 1 week	⑥
Cantello et al. (2007)	Italy	21/20	Total 28/17		Total 38.5 ± 13.3		Total 2,996 ± 148 months		A+B/A+C	Two sets of 500 reps at 30 s intervals for 26 weeks	⑥

Note: A: Conventional antiepileptic medication B: Low-frequency repetitive transcranial magnetic stimulation C: Sham stimulation (no stimulation) D: Antiepileptic medication with wake-up call injection ‘---’: not reported; ① Digit span forward and backward: Digit span forward and backward ② Simple reaction time: Simple reaction time ③ Stroop test: Stroop test ④ MoCA scale: Montreal Cognition Assessment Scale ⑤ MMSE: Measy Mental State Evaluation Scale ⑥ EEG.

### Quality assessment of the literature

The 12 studies included in this review (Cantello et al., 2007; Du & Zhao, 2021; Fregni et al., 2006; Wang, Zhao & Du, 2022; Zhao, Zheng & Ma, 2018; Hu et al., 2024; Geng, 2022; Lai et al., 2018; Hu et al., 2023; Huang, 2018; Zhang, 2020; Gao, 2020; Zhao, 2018) were all randomized controlled trials (RCTs). All studies met the criteria for random allocation, baseline comparability, intention-to-treat analysis, between-group statistical comparisons, and reporting point estimates with variability. One study met the criterion for allocation concealment, and two studies met the criterion for blinding of the outcome assessment. The PEDro scores ranged from 5 to 8, with a mean of 6.3. No low-quality studies were identified, indicating that the overall methodological quality was high. The details are provided in Table 4.

**Table 4:** Literature quality assessment.

Inclusion of studies	Eligibility criteria	Random allocation	Assignment hiding	Baseline similarity	Blindness of the study population	Therapist blindness	Results-based assessment of blindness	Participation rate >85 per cent	Intention-to-treat analysis	Analysis of statistical results between groups	Point measurements and difference values	Totals
Fregni et al. (2006)	1	1	1	1	1	0	0	1	1	1	1	8
Du & Zhao (2021)	1	1	0	1	0	0	0	1	1	1	1	6
Lai et al. (2018)	1	1	0	1	0	0	0	1	1	1	1	6
Hu et al. (2023)	1	0	0	1	0	0	0	1	1	1	1	5
Huang (2018)	1	1	0	1	0	0	0	1	1	1	1	6
Geng (2022)	1	1	0	1	0	0	0	1	1	1	1	6
Wang, Zhao & Du (2022)	1	1	0	1	0	0	1	1	1	1	1	7
Zhao, Zheng & Ma (2018)	1	1	0	1	0	0	0	1	1	1	1	6
Gao (2020)	1	1	0	1	0	0	0	1	1	1	1	6
Hu et al. (2024)	1	1	0	1	0	0	0	1	1	1	1	6
Zhao (2018)	1	1	0	1	0	0	0	1	1	1	1	6
Cantello et al. (2007)	1	1	0	1	1	0	1	1	1	1	1	8

### Meta-analysis results

#### Effect of LF-rTMS on cognition in patients with epilepsy

**Figure 2:**
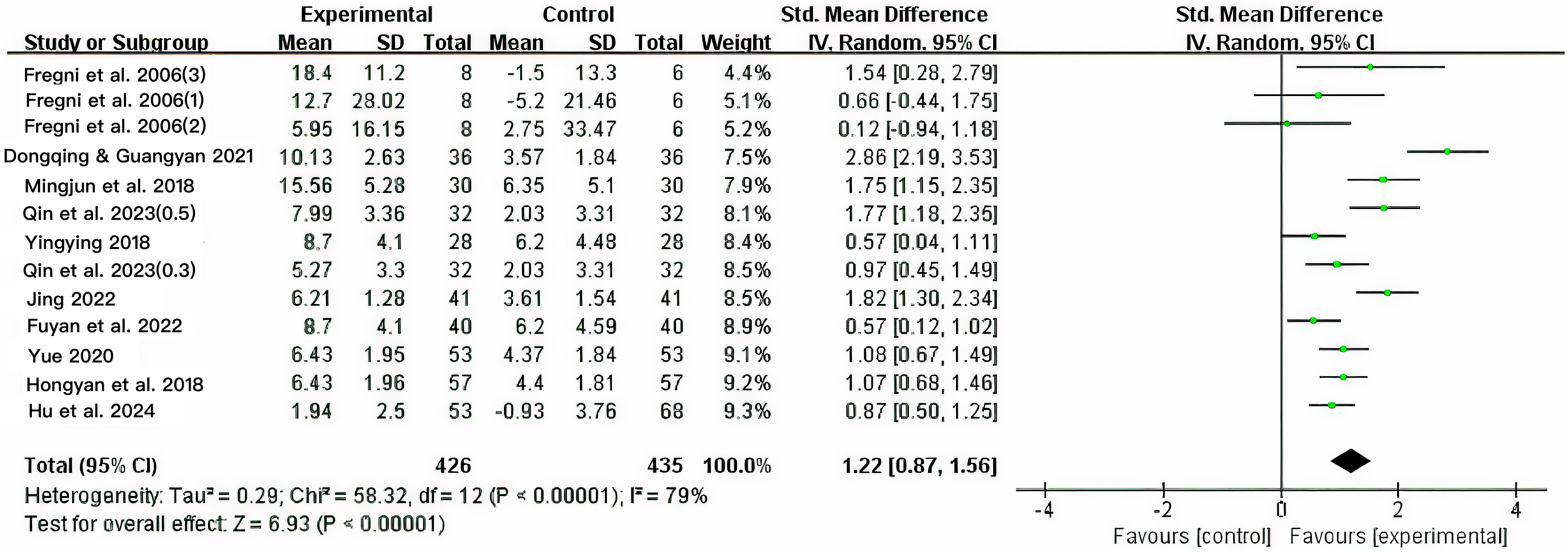
Combined total effect sizes for cognition functions (Fregni et al., 2006; Du & Zhao, 2021; Lai et al., 2018; Hu et al., 2023; Huang, 2018; Geng, 2022; Wang, Zhao & Du, 2022; Gao, 2020; Zhao, Zheng & Ma, 2018; Hu et al., 2024).

Ten of the 12 included studies (*n* = 861) compared low-frequency repetitive transcranial magnetic stimulation (LF-rTMS) with sham stimulation or conventional pharmacotherapy. As shown in Fig. 2, heterogeneity was high (I^2^ = 79%, *P* < 0.00001); therefore, a random-effects model was applied. The pooled effect size was significant (SMD = 1.22, 95% CI [0.87–1.56], *P* < 0.00001), indicating that LF-rTMS significantly improved cognitive function in patients with epilepsy compared with that in controls.

#### Effect of LF-rTMS on ED in epilepsy patients

**Figure 3:**
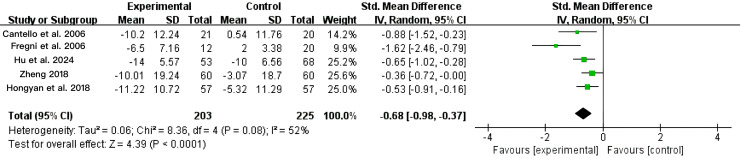
Combined total effect sizes for ED (Cantello et al., 2007; Fregni et al., 2006; Hu et al., 2024; Zhao, 2018; Zhao, Zheng & Ma, 2018).

As shown in Fig. 3, the meta-analysis indicated that low-frequency repetitive transcranial magnetic stimulation (LF-rTMS) significantly reduced abnormal epileptiform discharges in patients with epilepsy. Five of the 12 included studies (*n* = 428) compared LF-rTMS with sham stimulation or conventional pharmacotherapy. Heterogeneity analysis showed I^2^ = 52% and *P* = 0.08, indicating moderate heterogeneity; therefore, a random-effects model was applied. The pooled effect size was SMD = −0.68 (95% CI: [−0.98 to −0.37], *P* < 0.0001), indicating that LF-rTMS significantly reduced abnormal epileptiform discharges in patients with epilepsy compared to controls.

#### Subgroup analyses

Subgroup analyses of cognitive function and abnormal epileptiform discharges were conducted to explore the potential sources of heterogeneity (Table 5). The effects of low-frequency repetitive transcranial magnetic stimulation (LF-rTMS) on cognitive function and ED in patients with epilepsy may be influenced by factors such as age, intervention frequency, intervention duration, and session length.

**Table 5:** Meta-analysis results of LF-rTMS on cognition function in epilepsy patients.

Outcome indicator		Number of studies included	*P*-value	I^2^/%	Meta-analysis results	
					SMD (95%CI)	*P*-value
Cognitive functions		13 (865)		79	1.22 [0.87–1.56]	<0.0001
Age	<45 age	6 (240)	0.89	74	1.21 [0.52–1.73]	0.0003
	45–60 age	4 (372)		91	1.35 [0.60–2.11]	0.0004
	≥60 age	3 (249)		70	1.17 [0.56–1.68]	<0.00001
Stimulation frequency	1 Hz	4 (124)	0.99	70	1.09 [0.23–1.95]	0.01
	0.5 Hz	5 (461)		59	1.05 [0.74–1.36]	<0.00001
	0.3 Hz	3 (204)		79	1.07 [0.42–1.73]	0.001
Intervention duration	≤4 weeks	7 (439)	0.04	2	0.91 [0.71–1.11]	<0.00001
	>4 weeks	6 (422)		87	1.60 [0.97–2.23]	<0.00001
Session time	≤20 min/time	7 (400)	0.55	61	1.03 [0.65–1.42]	<0.00001
	>20 min/time	3 (273)		94	1.04 [0.27–2.54]	0.02
Frequency of intervention	>7 times/week	5 (212)	0.04	25	0.82 [0.45–1.19]	<0.0001
	≤7 times/week	8 (649)		85	1.43 [0.97–1.88]	<0.00001
Stimulus area	Cz central point	4 (122)	0.17	0	0.61 [0.24–0.98]	0.001
	Left and right temporal regions and the middle of the forehead	3 (276)		24	0.95 [0.66–1.24]	<0.00001
	Epileptogenic focus	3 (249)		70	1.17 [0.65–1.68]	<0.00001
Intervention drugs	Daily ASM treatment	4 (122)	0.10	0	0.61 [0.24–0.98]	0.001
	Levetiracetam	6 (471)		79	1.27 [0.82–1.71]	<0.00001
	Levetiracetam and wake-up call injection	2 (220)		0	1.07 [0.79–1.36]	<0.00001

##### Subgroup analysis of cognition in epilepsy patients

For cognitive function outcomes, age groups in the included studies were stratified into three categories: <44 years, 45–59 years, and ≥60 years; intervention frequency was categorized as 1, 0.5, or 0.3 Hz; intervention duration was classified as ≤4 weeks or >4 weeks; intervention duration per session was classified as ≤20 min or >20 min; intervention frequency (per week) was grouped as ≤7 sessions or >7 sessions; stimulation site was categorized as Cz (central point) or specific cortical targets (*e.g*., epileptogenic focus, temporal regions); and concomitant antiseizure medications (ASMs) were classified as standard therapy, levetiracetam monotherapy, levetiracetam combination therapy, or other regimens.

Subgroup analyses (Table 5) indicated that all the differences were statistically significant. Specifically, LF-rTMS showed greater effects on cognitive function in patients with epilepsy under the following conditions: age 45–60 years (I^2^ = 91%, SMD = 1.35, 95% CI [0.60–2.11], *P* = 0.0004); stimulation frequency of 1 Hz (I^2^ = 70%, SMD = 1.09, 95% CI [0.23–1.95], *P* = 0.01); session duration >20 min (I^2^ = 94%, SMD = 1.04, 95% CI [0.27–2.54], *P* = 0.02); intervention duration >4 weeks (I^2^ = 87%, SMD = 1.60, 95% CI [0.97–2.23], *P* < 0.00001); weekly frequency ≤7 sessions (I^2^ = 85%, SMD = 1.43, 95% CI [0.97–1.88], *P* < 0.00001); stimulation site at the epileptogenic focus (I^2^ = 70%, SMD = 1.17, 95% CI [0.65–1.68], *P* < 0.00001); and concomitant use of levetiracetam (I^2^ = 79%, SMD = 1.27, 95% CI [0.82–1.71], *P* < 0.00001). In terms of heterogeneity sources, subgroup analyses for intervention duration, intervention frequency, concomitant drug use, and stimulation site all showed I^2^ < 50%, indicating substantially reduced heterogeneity. These findings suggest that these factors may be the main sources of heterogeneity.

##### Subgroup analysis of ED in patients with epilepsy

For the outcome of abnormal epileptiform discharges, participants were stratified into two age subgroups: ≥45 and <45 years. Intervention duration was classified into two subgroups: ≤1 week and >1 week.

Subgroup analysis (Table 6) showed that LF-rTMS was more effective in reducing abnormal epileptiform discharges in patients with epilepsy when age was <45 years (I^2^ = 75%, SMD = −0.88, 95% CI [−1.57 to −0.18], *P* = 0.01) and when the intervention duration was ≤1 week (I^2^ = 73%, SMD = −0.75, 95% CI [−1.28 to −0.55], *P* = 0.005). When subgroup analyses of age and intervention duration reached statistical significance, potential sources of heterogeneity were considered in the analysis. Heterogeneity for both age and intervention duration was reduced to <50%, indicating a substantial decrease in heterogeneity. Therefore, age and intervention duration may be key sources of heterogeneity.

**Table 6:** Meta-analysis results of LF-rTMS on ED in patients with epilepsy.

Outcome indicator		Number of studies included	*P*-value	I^2^/%	Meta-analysis results	
					SMD (95%CI)	*P*-value
Epileptiform discharges		5 (428)		52	−0.68 [−0.98 to −0.37]	<0.0001
Age	≥45 age	2 (235)	0.45	0	−0.59 [−0.85 to −0.33]	<0.0001
	<45 age	3 (193)		75	−0.88 [−1.57 to −0.18]	0.01
Intervention duration	≤1 weeks	3 (273)	0.68	73	−0.75 [−1.28 to −0.22]	0.005
	>1 weeks	2 (155)		0	−0.62 [−0.94, −0.30]	0.0002

#### Meta-regression analysis

Meta-regression analyses were performed to examine the association between publication year, sample size, and mean patient age with observed heterogeneity. For cognitive outcomes, the model showed a Tau^2^ of 0.4742, with the covariates explaining 85.8% of residual heterogeneity. However, none of the examined factors—publication year (*P* = 0.250), sample size (*P* = 0.534), or mean age (*P* = 0.587)—significantly predicted effect size. Owing to the limited number of studies reporting epileptiform discharges (ED), meta-regression was not performed for this outcome. The complete results are presented in Table 7.

**Table 7:** Meta-regression analysis.

	Covariate	Regression coefficient	Standard error	*t*-value	*P*-value
Cognition function	Publication year	0.090	0.078	1.15	0.251
	Sample size	−0.006	0.001	−0.62	0.535
	Mean age	−0.015	0.027	−0.54	0.587

#### Sensitivity analysis

**Figure 4:**
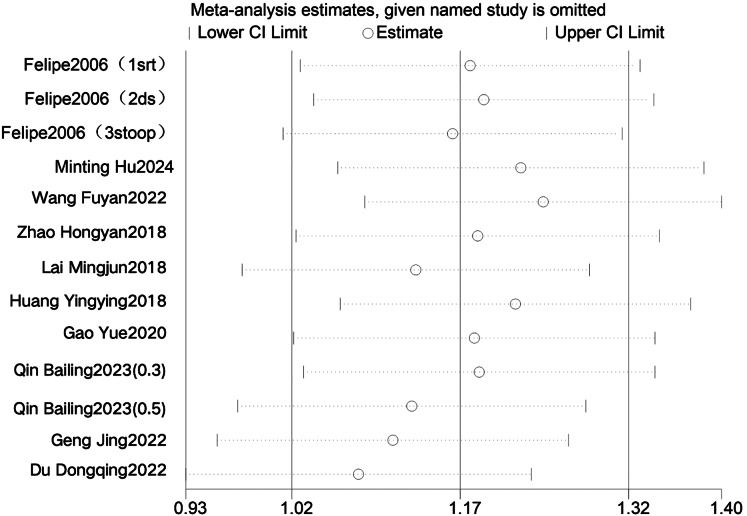
Sensitivity analysis of Cognition function (Fregni et al., 2006; Hu et al., 2024; Wang, Zhao & Du, 2022; Zhao, Zheng & Ma, 2018; Lai et al., 2018; Huang, 2018; Gao, 2020; Hu et al., 2023; Geng, 2022; Du & Zhao, 2021).

**Figure 5:**
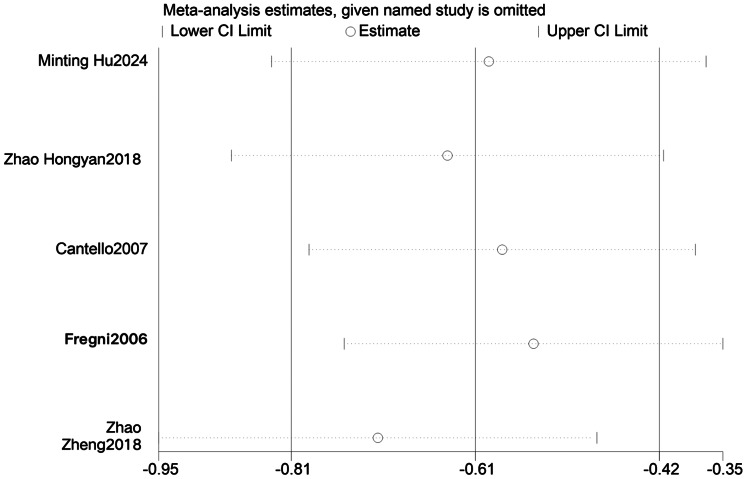
Sensitivity analysis of ED.

To determine whether individual studies contributed to between-study heterogeneity, we conducted sensitivity analyses of the effects of LF-rTMS on cognitive function and abnormal epileptiform discharges in patients with epilepsy (Figs. 4 and 5). The pooled effects were recalculated by sequentially excluding each study (Table 8). Excluding the study by Du & Zhao (2021) yielded a pooled effect on cognitive function of SMD = 1.09, 95% CI [0.81–1.36], *P* < 0.001, with I^2^ decreasing from 79% to 66%, although heterogeneity remained high. Exclusion of other individual studies resulted in pooled SMDs ranging from 0.81 to 1.64 and I^2^ values ranging from 78% to 81%, all with *P* < 0.001. Excluding the study by Fregni et al. (2006) yielded a pooled effect on ED of SMD = −0.55, 95% CI [−0.75 to −0.35], *P* < 0.001, with I^2^ decreasing from 52% to 0%, indicating a marked reduction in heterogeneity and a statistically significant difference compared with controls. The exclusion of other studies yielded pooled SMDs ranging from −0.78 to −0.65 and I^2^ values ranging from 49% to 64%, all with *P* < 0.001.

**Table 8:** Combined effects of excluding individual studies of cognition function and ED.

	Inclusion of studies	Effect size	95% CI	*P* (Merger effect)	I^2^/%
	Fregni et al. (2006) (1)	1.20	[0.84–1.56]	<0.001	81
	Fregni et al. (2006) (2)	1.25	[0.89–1.60]	<0.001	81
	Fregni et al. (2006) (3)	1.28	[0.93–1.62]	<0.001	80
	Du & Zhao (2021)	1.09	[0.81–1.36]	<0.001	66
Cognitive functions	Lai et al. (2018)	1.17	[0.81–1.53]	<0.001	80
	Hu et al. (2023) (1)	1.17	[0.81–1.53]	<0.001	80
	Huang (2018)	1.27	[0.91–1.63]	<0.001	79
	Hu et al. (2023) (2)	1.24	[0.86–1.61]	<0.001	81
	Geng (2022)	1.16	[0.81–1.51]	<0.001	79
	Wang, Zhao & Du (2022)	1.28	[0.92–1.64]	<0.001	78
	Gao (2020)	1.23	[0.84–1.61]	<0.001	81
	Zhao, Zheng & Ma (2018)	1.23	[0.84–1.62]	<0.001	81
	Hu et al. (2024)	1.25	[0.87–1.63]	<0.001	80
	Cantello et al. (2007)	−0.65	[−1.00 to −0.30]	0.0002	62
	Fregni et al. (2006)	−0.55	[−0.75 to −0.35]	<0.001	0
ED	Hu et al. (2024)	−0.72	[−1.15 to −0.30]	<0.001	64
	Zhao (2018)	−0.78	[−1.14 to −0.43]	<0.001	49
	Zhao, Zheng & Ma (2018)	−0.76	[−1.17 to −0.34]	<0.001	63

The study by Fregni et al. (2006) was the only one in which all participants had cortical developmental abnormalities and refractory epilepsy, suggesting that the disease type may have been a source of heterogeneity. After excluding this study, both the pooled SMD and I^2^ values remained relatively stable, indicating the robustness of the results. These findings indicate that LF-rTMS effectively reduced abnormal epileptiform discharges in patients with epilepsy compared with those in the controls.

### Publication bias

**Figure 6:**
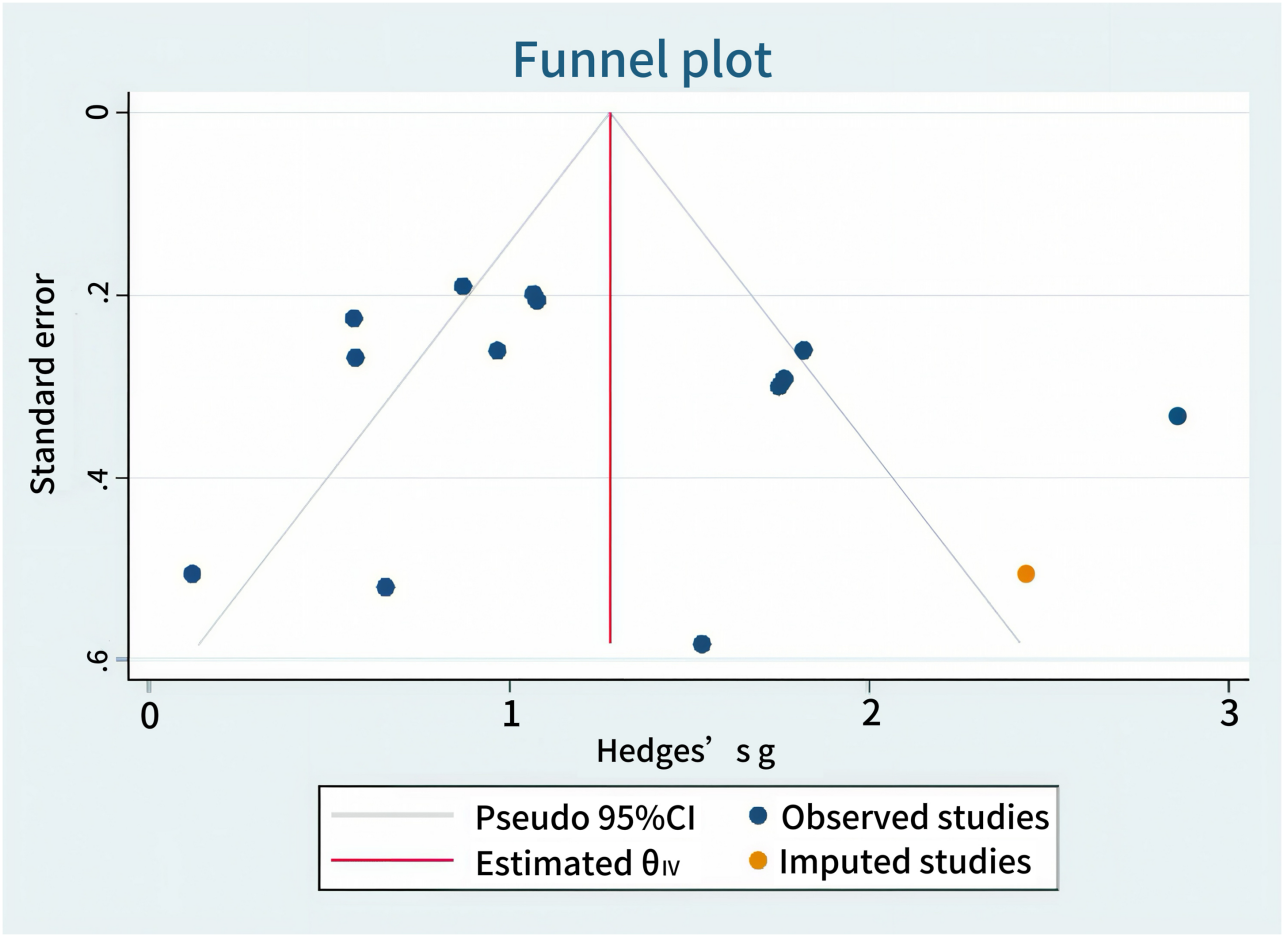
Cognition publication bias.

**Figure 7:**
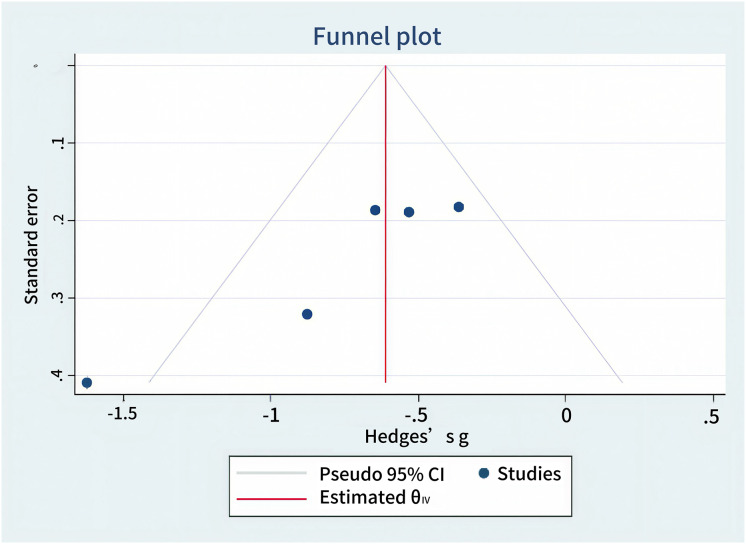
ED publication bias.

This study assessed the publication bias for cognitive function and abnormal epileptiform discharge outcomes. Egger’s test yielded *P* > |t| = 0.9427 (>0.05) for cognitive function and P > |t| = 0.0745 (>0.05) for ED, indicating no significant publication bias. Publication bias was further evaluated using the non-parametric trim-and-fill method, which showed no material change in effect sizes or confidence intervals before and after adjustment, confirming the absence of a significant publication bias. The results are illustrated in Figs. 6 and 7.

### Evaluation of the quality of evidence

**Figure 8:**
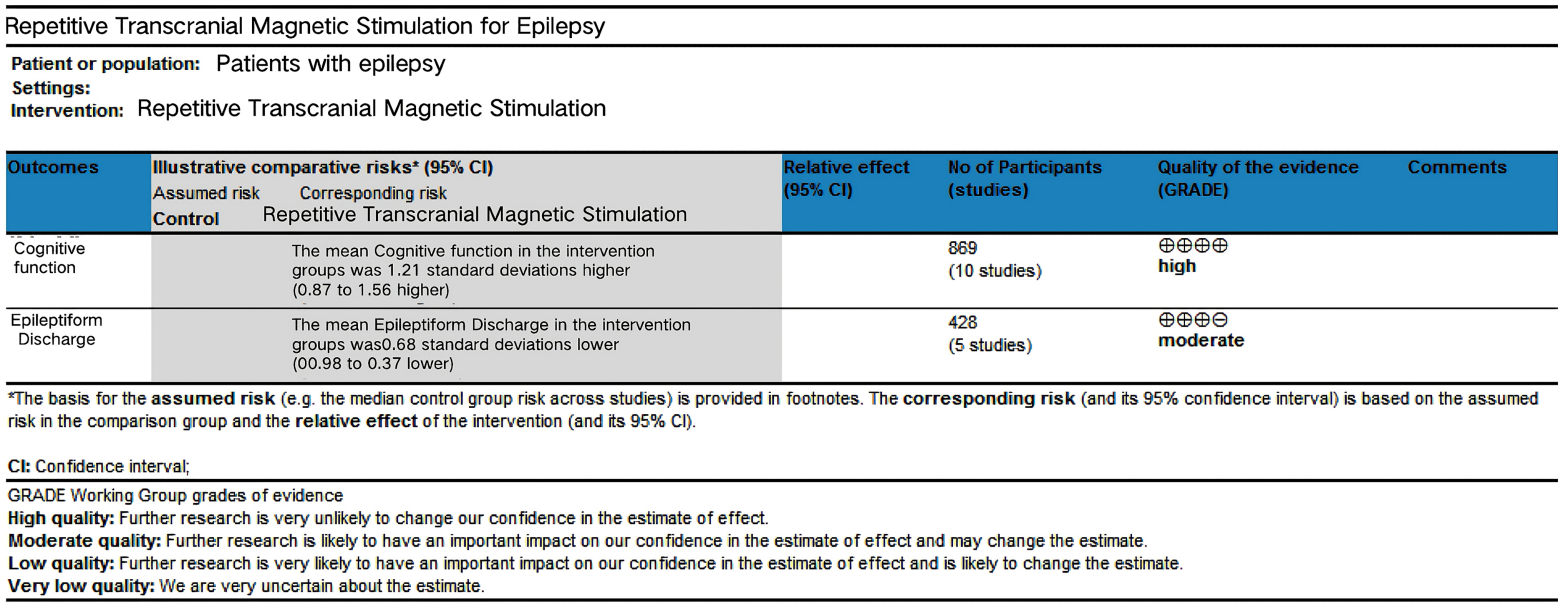
GRADE quality of evidence evaluation.

The GRADEpro assessment indicated that the quality of evidence was high for cognition and moderate for Epileptiform Discharges (ED), as detailed in Fig. 8. The evidence for ED was downgraded due to concerns regarding the risk of bias, primarily arising from a lack of blinding in specific studies. For instance, the studies by Zhao, Zheng & Ma (2018) and Zhao (2018) did not implement blinding of participants and personnel, which could have influenced the subjective components of ED assessment or intervention adherence. This limitation introduces a potential performance bias, justifying the downgrade to moderate quality.

## Discussion

The results of this study demonstrate that low-frequency repetitive transcranial magnetic stimulation (LF-rTMS) significantly improves cognitive function and reduces epileptiform discharges (ED) in patients with epilepsy, consistent with previous findings (Mishra et al., 2020; Wang et al., 2024). LF-rTMS showed greater cognitive benefits in patients aged 45–60 years, at a stimulation frequency of 1 Hz, with session durations >20 min, intervention durations >4 weeks, weekly frequency ≤7 sessions, stimulation targeted at the epileptogenic focus, and when combined with levetiracetam. LF-rTMS was more effective in reducing ED in patients <45 years of age and with intervention durations ≤1 week. LF-rTMS may modulate neuronal excitability, inhibit hyperactive neurons, enhance neuronal function and metabolism, and influence both local and remote cortical activity, thereby inducing beneficial biological effects (Jiang et al., 2022). These effects can improve neural network function and structure, alleviate epileptic symptoms, facilitate recovery of neurological and cognitive function, and ultimately enhance patients’ quality of life (Pang et al., 2022). In addition, LF-rTMS may reduce cortical excitability, which could underlie its effect in decreasing ED (Chen et al., 1997).

This study systematically reviewed 12 articles evaluating the effects of low-frequency repetitive transcranial magnetic stimulation (LF-rTMS) on cognitive function and epileptiform discharges (ED) in patients with epilepsy. The PEDro scale was used to assess study quality, yielding an average score of 6.3. No low-quality studies were identified, and the overall methodological quality was good. Limitations included downgrading factors: most studies did not adequately report blinding procedures or failed to implement allocation concealment, which may have influenced postintervention outcomes. Publication bias was assessed for cognition and ED, and no significant bias was detected. No significant downgrading was warranted for indirectness or imprecision in the evidence. The meta-analysis revealed substantial heterogeneity (I^2^ > 50%) for both cognition and ED outcomes. Subgroup analyses suggested that intervention duration, stimulation frequency, stimulation site, and concomitant drug use may have contributed to heterogeneity. Therefore, the effect of LF-rTMS on cognition was supported by high-quality evidence, whereas its effect on ED was supported by moderate quality evidence.

This study found that when the age is 45–60 years old, the intervention frequency of LF-rTMS is 1Hz, the session time is >20 min/session, the intervention duration is >4 weeks, the intervention frequency is ≤7 times/week, the stimulation site is at the epileptogenic focus, and the combined drug is levetiracetam, LF-rTMS has a better effect on the cognition of epilepsy patients. When the age is <45 years and the intervention duration is ≤1 week, LF-rTMS has a better effect on epileptic discharges in epilepsy patients.

The therapeutic efficacy of low-frequency repetitive transcranial magnetic stimulation (LF-rTMS) principally stems from its dual modulation of cortical excitability and synaptic plasticity, which collectively ameliorate both epileptiform discharges and cognitive deficits in patients with epilepsy. At frequencies ≤1 Hz, LF-rTMS suppresses pathological cortical hyperexcitability, normalizes aberrant neuronal firing patterns, and consequently attenuates ictal epileptiform discharges (Fregni, Schachter & Pascual-Leone, 2005; Xu, Lu & Li, 2024; Liu et al., 2024). Concurrently, LF-rTMS promotes synaptic repair and cortical restructuring by reducing excitatory synaptic transmission and the phosphorylation-dependent activation of plasticity-related proteins, including ribosomal protein S6, thereby reinforcing circuit stability (Fujiki, Yee & Steward, 2020; Li, Li & Pan, 2019; Sen, Capelli & Husain, 2018). Moreover, LF-rTMS exerts network-level effects by modulating the discrete neural circuits. Specifically, it dampens aberrant temporo-prefrontal projections, thereby relieving pathological inhibition of prefrontal cortical activity and normalizing higher-order cognitive processes, including memory formation and executive functioning (Fregni, Schachter & Pascual-Leone, 2005). Collectively, these mechanisms rectify the characteristic deficit in inhibitory control in epilepsy, reestablishing cortical excitability homeostasis, thereby concurrently suppressing epileptiform discharges and ameliorating cognitive impairment (Chou et al., 2022; Lissemore et al., 2019).

However, low frequency may reduce the therapeutic effect. LF-rTMS at 0.3 and 0.5 Hz can significantly reduce the rate of epileptic discharges. In addition, 0.5 Hz has a weaker stimulation ability on the bilateral frontal lobes of the brain than 1 Hz, and the correction ability of the neurotransmitter system is lower. Therefore, the improvement process of cognitive dysfunction in patients with epilepsy is relatively slow. Therefore, LF-rTMS at 1.0 Hz has a better effect on the recovery of cognitive function (Zhang, 2020). Currently, rTMS used in clinical practice takes 20 to 30 min to affect the functional area (Lefaucheur et al., 2020), so interventions with a stimulation duration of >20 min/session are more effective for cognition. Some scholars have found that levetiracetam treatment can reverse synaptic transmission defects and reduce abnormal electrical activity in the brain. Long-term levetiracetam treatment can improve memory and behavior, which may be why levetiracetam tablets are more effective in combination with other drugs (Sen, Capelli & Husain, 2018). This study also found that the improvement in cognitive function and ED in patients with epilepsy is affected by age. From an accelerated aging perspective, this may be due to the long-term accumulation of underlying lesions and continuous seizures or interictal epileptiform discharges caused by epilepsy itself. Consequently, the cognitive ability of patients with epilepsy continues to decline with age (Sen, Capelli & Husain, 2018). Therefore, age is a factor influencing cognitive function and ED. Some studies have shown that repeated and long-term stimulation can have a stronger and more lasting effect on patients with neurodegenerative diseases (Lin et al., 2019). Therefore, the LF-rTMS intervention on cognitive function may require long-term cumulative benefits to improve significantly. As this study only analyzed the time domain of epileptiform discharge based on the stimulating effect of LF-rTMS, the short-term intervention effect was more obvious. The time-domain analysis of epileptiform discharges in this study suggests that the suppressive effect of LF-rTMS manifests rapidly, with diminishing returns observed beyond the initial week as the response plateaus. In contrast, improvements in cognitive function likely depend on the cumulative neuroplastic effects of repeated stimulation, which develop over a longer period (Yuan et al., 2024). This fundamental difference in the temporal dynamics of the response—a rapid initial effect for ED *vs*. a gradual accumulation for cognition—explains the distinct optimal intervention durations.

## Limitations

This study has several limitations that merit careful consideration. First, substantial clinical heterogeneity, potentially stemming from unreported antiseizure medication schedules, unclassified epilepsy syndromes, and diverse age distributions, constrains the generalizability of our conclusions. Second, the limited number of studies reporting on epileptiform discharges resulted in only moderate certainty of evidence, necessitating cautious interpretation of these findings. Third, reliance on digitized data extraction from figures in a subset of studies introduces a potential source of measurement error. Finally, the restriction of our analysis to short-term outcomes precludes the assessment of the sustained therapeutic effects of LF-rTMS. Future large-scale, multicenter randomized trials with extended follow-up periods are required to validate these observations and determine the long-term clinical utility of rTMS.

## Conclusion

In summary, low-frequency repetitive transcranial magnetic stimulation (LF-rTMS) significantly improves cognitive function and reduces epileptiform discharges in patients with epilepsy. The efficacy of LF-rTMS is influenced by factors such as the intervention duration, stimulation frequency, and site. These findings provide evidence to guide clinical rehabilitation and improve the quality of life of patients. Current evidence supports a stimulation frequency of 1 Hz, session duration of >20 min, intervention duration of > 4 weeks, ≤7 sessions per week, and targeting the epileptogenic focus to optimize cognitive outcomes in epilepsy. For epileptiform discharges, LF-rTMS administered for ≤1 week may yield the most immediate benefits, although long-term advantages are evident.

## Supplemental Information

10.7717/peerj.20637/supp-1 Supplemental Information 1 Raw data.

10.7717/peerj.20637/supp-2 Supplemental Information 2 PRISMA Checklist.

10.7717/peerj.20637/supp-3 Supplemental Information 3 Intended audience.

## Additional Information and Declarations

### Data Availability

The following information was supplied regarding data availability:

This is a literature review.
