# Supplementary material for: Systematic reviews of low-frequency repetitive transcranial magnetic stimulation on cognition and epileptiform discharge in patients with epilepsy
Source: PeerJ. 2026 Feb 9;14:e20637. doi: 10.7717/peerj.20637 (PMC12897352; doi:10.7717/peerj.20637)
Supplement: Supplemental Information 1 [file peerj-14-20637-s001.docx]

| research | Mean | SD | Sample size of the experimental group | Mean | SD | Sample size of the control group |
| --- | --- | --- | --- | --- | --- | --- |
| Felipe2006（1srt） | 12.70 | 28.02 | 8 | -5.20 | 21.46 | 6 |
| Felipe2006（2ds） | 5.95 | 16.15 | 8 | 2.75 | 33.47 | 6 |
| Felipe2006（3stoop） | 18.40 | 11.20 | 8 | -1.50 | 13.30 | 6 |
| Minting Hu2024 | 1.94 | 2.50 | 53 | -0.93 | 3.76 | 68 |
| Wang Fuyan2022 | 8.70 | 4.10 | 40 | 6.20 | 4.59 | 40 |
| Zhao Hongyan2018 | 6.43 | 1.96 | 57 | 4.40 | 1.81 | 57 |
| Lai Mingjun2018 | 15.56 | 5.28 | 30 | 6.35 | 5.10 | 30 |
| Huang Yingying2018 | 8.70 | 4.10 | 28 | 6.20 | 4.48 | 28 |
| Gao Yue2020 | 6.43 | 1.95 | 53 | 4.37 | 1.84 | 53 |
| Qin Bailing2023（0.3） | 5.27 | 3.30 | 32 | 2.03 | 3.31 | 32 |
| Qin Bailing2023（0.5） | 7.99 | 3.36 | 32 | 2.03 | 3.31 | 32 |
| Geng Jing2022 | 6.21 | 1.28 | 41 | 3.61 | 1.54 | 41 |
| Du Dongqing2021 | 10.13 | 2.63 | 36 | 3.57 | 1.84 | 36 |

**Cognition**

**ED**

| research | Mean | SD | Sample size of the experimental group | Mean | SD | Sample size of the control group |
| --- | --- | --- | --- | --- | --- | --- |
| Minting Hu2024 | 53 | -14.00 | 5.57 | 68 | -10.00 | 6.56 |
| Zhao Hongyan2018 | 57 | -11.22 | 10.72 | 57 | -5.32 | 11.29 |
| Cantello2007 | 21 | -10.20 | 12.24 | 20 | 0.54 | 11.76 |
| Freni2006 | 12 | -6.50 | 7.16 | 20 | 2.00 | 3.38 |
| zhao zheng2018 | 60 | -10.01 | 19.24 | 60 | -3.07 | 18.70 |
