# Supplementary material for: Systematic reviews of low-frequency repetitive transcranial magnetic stimulation on cognition and epileptiform discharge in patients with epilepsy
Source: PeerJ. 2026 Feb 9;14:e20637. doi: 10.7717/peerj.20637 (PMC12897352; doi:10.7717/peerj.20637)
Supplement: Supplemental Information 3 [file peerj-14-20637-s003.docx]

The primary audience for this article includes clinical practitioners (such as neurologists and rehabilitation specialists) and academic researchers. Its content balances practical clinical applicability with scientific rigor, making it particularly suitable for professionals who rely on evidence-based approaches to develop treatment strategies or conduct related research
